# The Detection of *Chlamydia pneumoniae*, *Helicobacter pylori* and *Cytomegalovirus* in Non-Atherosclerotic Arteries of Patients with Coronary Artery Disease

**DOI:** 10.3390/pathogens13110927

**Published:** 2024-10-25

**Authors:** Dalila Šačić, Uroš Tomić, Jelena Milašin, Svetozar Putnik, Milena Jovanović, Sanja Radojević Škodrić, Sofija Glumac

**Affiliations:** 1 Cardiology Department, University Clinical Center of Serbia, 11000 Belgrade, Serbia; 2 Belgrade Medical Faculty, University of Belgrade, 11000 Belgrade, Serbia; svetozar073@yahoo.com; 3 Clinic for Periodontology and Oral Medicine, School of Dental Medicine, University of Belgrade, 11000 Belgrade, Serbia; urostomic27@gmail.com; 4 Department of Human Genetics, School of Dental Medicine, University of Belgrade, 11000 Belgrade, Serbia; jelena.milasin@stomf.bg.ac.rs; 5 Cardiac-Surgery Department, University Clinical Center of Serbia, 11000 Belgrade, Serbia; 6 Department of Pathology, Faculty of Medicine, University of Belgrade, 11000 Belgrade, Serbia; milenaj300@gmail.com (M.J.); sanjaskodric@gmail.com (S.R.Š.)

**Keywords:** coronary artery disease, atherosclerosis, infection, inflammation, *Chlamydia pneumonia*, *Helicobacter pylori*, *Cytomegalovirus*, aorta, internal mammary artery

## Abstract

Atherosclerotic coronary artery disease (ACAD) is a major cause of global morbidity and mortality, characterized as an inflammatory process due to damage to blood vessel walls by risk factors like aging, hyperlipidemia, hypertension, smoking, and diabetes. Infectious agents, including *Chlamydia pneumoniae* (Cpn), Cytomegalovirus (CMV), and *Helicobacter pylori* (HP), have been implicated in ACAD’s pathophysiology. A study with 56 subjects undergoing coronary artery bypass grafting (CABG) aimed to detect Cpn, CMV, and HP DNA in unaffected artery segments and explore associations with disease progression and inflammation markers. The study found infectious agents’ DNA in 21.4% of samples, HP in eight samples, and CMV and Cpn in four samples each. Significant correlations were observed between HP and overweight or obese subjects, as well as between the presence of infectious agents and inflammation marker values. An association between HP and renal function was also noted. The findings reaffirm previous discoveries of infectious agents in non-clinically affected arteries used as CABG grafts. Correlations identified between the presence of HP, CMV, and Cpn DNA in grafts and several biomarkers of inflammation and obesity emphasize the potential role of these infectious agents in ACAD pathogenesis.

## 1. Introduction

Cardiovascular diseases (CVD) are the primary cause of death globally, resulting in an estimated 17.9 million fatalities annually [1]. The most common type of CVD is atherosclerotic CVD (ASCVD).

Although classical risk factors, non-modifiable risk factors (like age, gender, ethnicity, and family history), and modifiable risk factors (like high blood lipids, high blood pressure, smoking, obesity, diabetes, poor diet, and a sedentary lifestyle) are known to contribute to coronary artery disease (CAD), its complete etiology remains unclear [2]. In recent years, traditional modifiable risk factors have been effectively reduced, yet the incidence of CAD remains high [3]. Additionally, more than 30% of patients with atherosclerosis lack these classical risk factors, suggesting the involvement of unknown factors in its pathogenesis.

Atherosclerosis is nowadays seen as an inflammatory process resulting from chronic damage of the blood vessel wall by various risk factors such as aging, hyperlipidemia, hypertension, smoking, diabetes mellitus, and exposure to certain infectious agents [4]. Prolonged exposure to oxidative stress activates multiple cell types involved in inflammation, including leukocytes, platelets, endothelial cells, and smooth muscle cells, leading to chronic inflammation of the vessel wall, a crucial pathophysiological mechanism of atherosclerosis [5].

The relationship between atherosclerosis, ASCVD and infections has been extensively studied, with evidence indicating that specific infections pose a risk for the development and/or worsening of ASCVD. Notably, associations of ASCVD with periodontal disease, *Helicobacter pylori* (HP), Cytomegalovirus (CMV), *Chlamydia pneumoniae* (Cpn), human immunodeficiency virus (HIV), herpes simplex virus (HSV), and COVID-19 (SARS-CoV-2) infection have been described [6,7,8,9,10,11,12]. However, the mechanisms of these associations remain unclear, and research findings have been controversial.

*Helicobacter pylori* is a common Gram-negative bacterium, and it is estimated that almost half of the human population has been affected by this microorganism [13]. Most individuals are clinically asymptomatic, but a noticeable fraction can develop gastrointestinal disorders, ranging from chronic gastritis to gastric cancer. HP infection has also been closely linked to the development of autoimmune disorders, idiopathic thrombocytopenia, and diabetes mellitus [14].

Several meta-analysis studies have shown that HP infection increases the odds of ASCVD. Patients infected with HP expressing the virulence factor cytotoxin-associated gene A (CagA) had greater coronary artery stenosis and restenosis after percutaneous transluminal coronary angioplasty [15]. Furthermore, early HP eradication therapy led to a significant decrease in coronary heart disease complications, especially in patients below 60 years of age [16]. Despite many efforts and studies, there are still conflicting results on the possible role of HP infection in atherosclerosis. Most of the studies on HP’s effect on cardiovascular disorders were based on serological diagnosis of HP infection (plasma anti-*H. pylori*, anti-CagA IgG) or rapid urease tests. Still, some studies tried to identify the presence of HP DNA in atherosclerotic and non-atherosclerotic arterial segments [8]. HP infection can cause an elevation in inflammation biomarkers (CRP, platelet-to-lymphocyte ratio, neutrophil-to-lymphocyte ratio), which are associated with the progression of ASCVD [17].

Cytomegalovirus (CMV), a DNA virus belonging to the herpes virus family, is widely distributed in the population [18,19]. Several studies have provided evidence linking cytomegalovirus infection to the development of atherosclerosis. Researchers have detected CMV DNA in atherosclerotic plaques and have found a correlation between CMV presence and restenosis in patients who have undergone coronary atherectomy or angioplasty [20,21,22].

A meta-analysis involving 34,564 participants (including 4789 ASCVD patients) demonstrated a 22% increase in the relative risk of ASCVD due to CMV infection [22]. In a group of 12,903 cases and 16,097 controls, patients with positive CMV IgG had a 1.7 times higher risk of ASCVD, while those with positive CMV IgM had almost a 3 times higher risk of CAD [23]. CMV infection is associated with a systemic inflammatory reaction, leading to elevated inflammatory biomarkers (CRP, IL-1, IL-6, TNF, IFN) that can contribute to the progression of CAD [24]. CMV infection impairs endothelial function through direct infection of endothelial and smooth muscle cells of blood vessels, followed by dysregulation of nitric oxide production and a local inflammatory response [17].

*Chlamydia pneumoniae* (Cpn) is a common cause of respiratory infections, ranging from upper respiratory to severe pneumonia, with a seroprevalence of more than 50% that increases with age. Cpn infection is related to atherosclerosis, arthritis, and Alzheimer’s dementia, with serological evidence, DNA, or whole bacteria detection in atherosclerotic plaques, vascular smooth muscle cells, macrophages, and foam cells. In vivo and in vitro studies have shown that Cpn infection can stimulate atherogenesis through arterial wall infection, inflammation, intracellular production of reactive oxygen species, LDL oxidation, and accumulation and creation of foam cells [25,26,27].

The inflammatory nature of atherosclerosis is evidenced by the correlation of inflammatory markers, especially C-reactive protein (CRP), with the onset and progression of the disease [28]. An elevated erythrocyte sedimentation rate (ESR) may be an important indicator of coronary artery disease, possibly linked to its inflammatory condition [29]. Epidemiological studies have consistently shown that fibrinogen levels are a strong primary and secondary risk factor for coronary artery disease, cerebral and peripheral arterial disease, and are associated with the prevalence of arterial disease in these three sites. Additionally, epidemiological studies suggest that fibrinogen may serve as an important link between genetic and environmental influences on arterial disease [30].

Previous studies have shown that infection with HP, Cpn and CMV could be associated with the inflammatory component of atherosclerosis in coronary arteries, and have shown presence of infectious agents in atherosclerotic plaques, but also in non-atherosclerotic segments of coronary and other arteries, used as grafts in cardiovascular surgery, implying a possible relationship of infection and pre-atherosclerotic changes in arteries, such as endothelial dysfunction.

This study’s aim was to detect CMV, HP and Cpn DNA presence in non-atherosclerotic segments of arteries used as grafts in revascularization surgery, and to explore a possible relationship of these pathogens with the inflammatory component of atherosclerosis and the severity of atherosclerosis in patients with CAD.

## 2. Materials and Methods

Participants from the group of patients with coronary artery disease were selected based on inclusion criteria for the study, among patients hospitalized at the Clinic for Cardiovascular Surgery, University Clinical Centre of Serbia. Inclusion criteria for patients were age greater than or equal to 40 years at the time of signing the informed consent, diagnosis of coronary artery disease confirmed by coronary angiography, and no acute infection or inflammation. Each participant received an informed consent form before being enrolled in the study, presented by the physician-researcher. All participants who signed the informed consent form, after confirming their understanding of its contents, were included in the research.

The main exclusion criterion was coronary angiography showing stenosis lower than 50%.

Demographic data and biochemical data were obtained by the physician-researcher from the medical records (medical history) of the participants at the Clinic for Cardiovascular Surgery, University Clinical Centre of Serbia, adhering to standard measures for the protection of participants’ data. All of the analyzed blood samples were taken during the initial cardiologist and/or cardiovascular surgeon consultation while defining the type of surgical procedure appropriate for the patient treatment.

Small samples of distal parts of diagonal branches (branches of the left anterior descending coronary artery—LAD), parts of the internal mammary artery (Latin: *a. mammaria interna*) used for bypass grafting, and parts of the aorta without clinically assessed atherosclerotic degeneration were taken. Samples were collected during cardiothoracic surgeries (surgical myocardial revascularization bypass). Samples of the distal part of the diagonal branch, internal mammary artery, and aorta were taken from the patients. Immediately after the biopsy, the samples were placed in formalin bottles and transported within 6–48 h to the Institute of Pathology.

Tissue samples were placed in sterile tubes immediately after biopsies and transferred to the laboratory. The whole isolation procedure was performed in strictly aseptic conditions in the specially designed part of the laboratory reserved for DNA and RNA isolations. The laboratory is sanitized and UV disinfected daily.

Further tissue processing involved fixation, dehydration, and molding of samples in paraffin, followed by making paraffin sections for the isolation of patients’ entire deoxyribonucleic acid (DNA). After the isolation procedure, detection of the possible presence of HP, Cpn, and CMV in patient samples was carried out using the polymerase chain reaction (PCR) method.

### 2.1. DNA Isolation

DNA isolation from samples was performed using the phenol-chloroform method. Paraffin-embedded samples were cut on a microtome and afterwards xylene deparaffinized. DNA concentration and isolate pureness were measured using a spectrophotometer (BioSpec Nano, Shimadzu, Tokyo, Japan). Samples were stored at −20 °C until further examination.

### 2.2. Real-Time Polymerase Chain Reaction (qPCR)

Microorganism presence and quantification were determined using the *qPCR* method. The glyceraldehyde 3-phosphate dehydrogenase (GAPDH) gene was amplified for every sample in order to calculate relative bacterial/viral quantification. The final GAPDH qPCR mixture of 15 µL contained 20 ng of a DNA template, 0.5 µM primer, 0.25 µM TaqMan probe, 1× FastGene^®^ Probe qPCR Universal Mix (NIPPON Genetics EUROPE, Düren, Germany), and sterilized nuclease-free water. The temperature profiles were the following: 10 min at 95 °C, followed by 45 cycles of 15 s at 95 °C and 30 s at 58 °C.

The FastGene ICGreen 2× PCR Universal Mix (NIPPON Genetics EUROPE, Germany) was used to amplify the segments of HP, Cpn and CMV using the corresponding primers. The *qPCR* protocol included a holding stage at 95 °C for 3 min, followed by a cycling stage consisting of 45 cycles at 95 °C for 30 s, 60 °C for 30 s, and 72 °C for 30 s. Every qPCR run was conducted using the CFX96 real-time system (Bio-Rad Laboratories, Hercules, CA, USA) and followed by melting curve analysis to verify the amplification specificity. The qPCR mix of 10 µL contained 20 ng of a DNA template, 0.25 µM of the forward and reverse primer, the FastGene ICGreen 2× PCR Universal Mix (NIPPON Genetics EUROPE, Düren, Germany), and sterilized nuclease-free water All samples were tested twice. Sequences of the mentioned primers are listed in Table 1.

The outcomes were determined in terms of threshold cycle (Ct) values, and relative quantification was estimated using the ∆∆Ct technique [31]. The average value of duplicate samples was determined, and the relative quantification was established by comparing each microorganism value to the expression of glyceraldehyde 3-phosphate dehydrogenase.

### 2.3. Statistical Analysis

Depending on the type of variables and the normality of the distribution, results were presented as frequency (percent), median (range), and mean ± SD. The methods used for testing statistical hypotheses included *t*-test, Mann-Whitney test, chi-square test, and Fisher’s exact test. The correlation between variables was estimated using Pearson’s or Spearman’s correlation coefficient. Regression analysis was employed to analyze outcomes and potential predictors. Statistical hypotheses were analyzed at a significance level of 0.05. Statistical data analysis was performed using IBM SPSS Statistics 24 (IBM Corporation, Armonk, NY, USA).

## 3. Results

Most of the subjects were male (85.7%), over 60 years of age, overweight or obese (66%), non-smokers or ex-smokers, and admitted to the hospital due to non-stable angina pectoris (Table 2).

Main comorbidities were arterial hypertension and hyperlipoproteinemia (94.6%), followed by mitral regurgitation (78%), tricuspid regurgitation (50%) and congestive heart disease (48%). Diabetes mellitus was previously diagnosed in 33.9%, and chronic kidney disease stage 2 or 3 in 19.6% of the subjects.

Laboratory parameters showed increased markers of inflammation, such as C-reactive protein, fibrinogen and erythrocyte sedimentation rate (Table 3).

**Figure 1:**
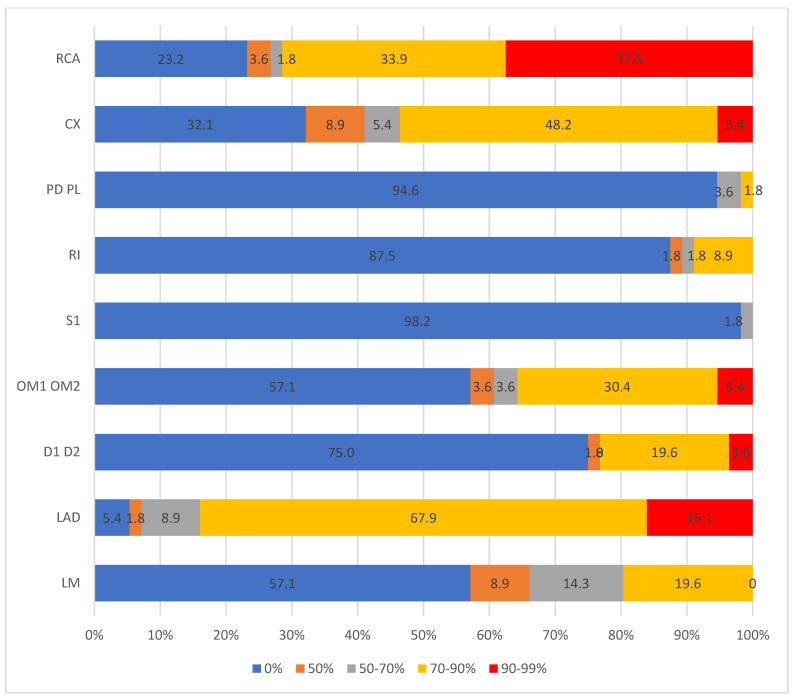
Location and the level of stenosis of coronary arteries in study sample. RCA: Right coronary artery; CX: Circumflex artery; PD PL: Posterior descending/posterolateral artery; RI: Ramus intermedius; S1: segment 1; OM: Obtuse marginal artery; D: Diagonal branch; LAD: Left anterior descending artery; LM: Left main artery.

Stenosis of the coronary arteries was most often found in LAD (94.6% of cases), with 45 subjects having three or four coronary arteries affected. The most significant changes were seen in RCA (37.5% subjects had stenosis over 90%) and LAD (16.1% subjects had stenosis over 90%) (Figure 1).

Sampling and PCR analysis of the non-atherosclerotic arterial segments identified the presence of microorganism DNA in 12 subjects (21.4%). HP was found in eight samples (four from the coronary artery, four from the internal mammary artery), Cpn was found in four samples (two from the coronary artery, two from the internal mammary artery), and CMV was discovered in four samples (one from the coronary artery, three from the internal mammary artery). In two patients, PCR showed simultaneous presence of two different pathogens, while in one subject, all three pathogens were found.

After coronary artery bypass graft surgery, 57.1% of the subjects had some postoperative complications that usually appeared in the early recovery phase (48.2%). Pleural effusion was diagnosed in 16 subjects (28.6%) and atrial fibrillation in 11 subjects (19.6%). In three cases, there was a need for re-intervention, and fatal outcomes happened in one subject.

There was no significant correlation between BMI, or BMI category, and level of stenosis in different segments of affected coronary arteries (Pearson’s or Spearman’s correlation coefficient, *p* > 0.05).

Independent samples tests showed no significant relationship between BMI difference and presence of HP (*p* = 0.144), CMV (*p* = 0.996), or Cpn (*p* = 0.253). When analyzed by BMI category, presence of infectious agents was seen more in overweight and obese subjects, but statistical significance was reached only in the case of HP (*p* = 0.044).

Presence of HP was significantly and positively correlated with fibrinogen level, and negatively correlated with eGFR. On the other hand, Cpn was negatively correlated with both fibrinogen and ESR levels, as well as urea level. CMV presence was positively correlated with fibrinogen and ESR (Table 4). Presence of infectious agents had no significant influence on CRP, lipid status or any of the elements of the complete blood count.

In order to exclude possible confounders, a linear regression was performed with presence of microorganisms as the independent variable and laboratory parameters as the dependent variable, controlled for age, sex and BMI. The previously recognized correlations persisted in this model (Table 5).

The authors performed a quantitative PCR study; the statistical analysis of absolute values confirmed the same correlations as the analysis following distribution by category (microorganism present/not present).

## 4. Discussion

This study identified presence of HP, Cpn and CMV in segments of coronary and internal mammary arteries, used for coronary artery bypass surgery in patients with significant coronary artery disease. Presence of HP was positively correlated with fibrinogen level, and negatively correlated with eGFR. Furthermore, Cpn was negatively correlated with both fibrinogen and ESR levels, and positively correlated with urea level. Identified CMV positively correlated with fibrinogen, ESR and urea.

The results of the study have strengthened the assumption that atherosclerosis is basically a chronic inflammatory process [4,5], as well as the idea that infectious agents can have a notable impact on the development and progression of atherosclerosis [17].

Previous studies have provided evidence that HP, Cpn and CMV are significantly associated with the progression of atherosclerotic cardiovascular diseases, most of all coronary artery disease [9].

Researchers have initially focused on indirect detection of pathogens (specific IgM and IgG antibodies in bodily fluids), in the acute or chronic infection, and later on, PCR and other techniques have proven the presence of microbial DNA directly in atherosclerotic plaques, and to a lesser extent in arterial segments with no clinically detectable atherosclerotic lesions.

In our study, HP DNA was found in 14%, CMV in 7%, and Cpn in 7% of the subjects, with similar distribution in coronary arteries and internal mammary arteries, but not in aortic segments.

In a large meta-analysis of 44 studies on the prevalence of microorganisms in atherosclerotic plaques of coronary arteries in patients with CAD, HP was recognized in 19%, CMV in 29% and Cpn in almost 43% of atheromas [32]. The meta-analysis from 2020 revealed that the prevalence of Cpn, HP, and CMV in patients with CAD were 25.1%, 2.8%, and 64.4%, respectively [9]. Kowalski et al. identified HP in atherosclerotic lesions of 48% patients with CAD [15].

Reszka et al. analyzed samples of non-atherosclerotic aortic segments in patients with CAD in comparison to patients with aortic valve disease and found very high prevalence of HP DNA in both groups (80% vs. 85%), which led them to the conclusion that the presence of HP in apparently healthy aorta segments was not significant for CAD. Similarly, they identified Cpn in 27.5% of samples [33].

Iriz et al. analyzed biopsy samples taken from aorta and internal mammary artery for CABG in patients with CAD. Real-time PCR showed that 26% of these patients had HP DNA in aortic, but not in internal mammary artery segments, compared to zero patients in the control non-CAD group. Most of the HP-positive patients required emergency intervention and had elevated CRP, blood leucocyte count, cholesterol, and LDL-C and HDL-C cholesterol in comparison to HP-negative patients and controls [34]. In another study from the same research group [35], Cpn DNA was found in the affected aorta regions, but not in the healthy mammary arteries. The authors assumed that the internal mammary artery was not affected due to the potential resistance to atherosclerosis attributed to elevated nitric oxide synthetase, angiotensin-converting enzyme and endothelin A receptor expression in this artery.

Izadi et al. came to a similar result, with 29.5% of atherosclerotic plaques being HP DNA positive, while internal mammary segments from the same patients had no HP DNA identified [36]. Their second research study focused on CMV DNA detection in patients with acute coronary syndrome, and detected it in 27% of the atherosclerosis specimens, but not in the normal mammary arteries [21].

Kilic et al. found HP DNA in 48.2% of atherosclerotic samples and in 19.6% of healthy vascular samples, with higher rates in atheromatous coronary arteries and aorta, but similar rates in plaque and non-plaque carotid arteries. They also found high rates of CMV in both types of samples, with no significant difference [37].

Heybar et al. [38] detected CMV DNA in 14.5% of the atherosclerotic aortic samples from CAD patients and 4% of the non-atherosclerotic aorta samples from controls, which suggested that CMV was associated with a high risk of atherosclerosis. No CMV DNA was detected in healthy aorta samples in CAD patients. A meta-analysis demonstrated that the positive rates of CMV IgG, IgM, genomic DNA, and protein pp65 were higher in the atherosclerosis subjects [39].

Taylor-Robinson et al. [40] analyzed the presence of Cpn DNA in atherosclerotic and non-affected arterial and venous segments, and found Cpn presence only in the affected blood vessels. Adiloglu et al. [41], on the other hand, showed HP DNA in 21% of coronary atherosclerotic plaques vs. 7% in healthy internal mammary segments, showing that this artery is not completely safe from HP infection, which is in line with the results in our study. Similarly, Pucar et al. [42] analyzed segments of coronary arteries with atherosclerosis and internal mammary arteries without atherosclerotic degeneration in the same subjects for presence of putative pathogens, CMV and Cpn, and found that CMV DNA was present in 67% of affected arteries, but in 47% of non-affected arteries. Cpn DNA was more present in healthy (40%) vs. non-healthy arteries (33%).

A recent study [43] that involved healthy adult subjects with or without HP infection (rapid urease test) used cardiac computed tomography to identify subclinical coronary artery atherosclerotic lesions and showed that HP-positive individuals had a three-fold greater odds of having early atherosclerotic lesions than HP negative subjects.

One possible explanation is connected to the effects of HP on endothelial dysfunction through increased oxidative stress, inflammation, decreased NO formation, cytokines, changes in lipid and glucose metabolisms, and exosome-mediated pathways. It is plausible that HP presence in the non-atherosclerotic segments of arteries could be a predictor of future endothelial dysfunction and atherosclerosis [44,45].

In our study, subjects where presence of HP, CMV, and Cpn was confirmed were usually overweight or obese, but only for HP-positive patients did the relationship reach statistical significance. This is in line with previous studies where HP was positively correlated with BMI [46]. Furthermore, none of the identified agents showed a significant effect on the lipid profile of our subjects. In a large meta-analysis published in 2022, Cpn infection was related to higher triglycerides, but not to cholesterol, HDL-C or LDL-C in patients with CAD [47].

In this study, we showed that HP and CMV presence was significantly and positively linked to fibrinogen and ESR, while Cpn was negatively linked, but the correlation with CRP did not reach statistical significance. Previous studies yielded conflicting results, with some showing a clear correlation, and some, like ours, with no significant influence on CRP [27,35,45,48].

When it comes to fibrinogen, in a study by Yusuf et al. [49], HP infection was associated with higher fibrinogen levels in older patients with ischemic heart disease, and HP eradication treatment led to normalization of fibrinogen levels. Similar results and recommendations were proposed by Kounturas et al. [50].

Our study also revealed a significant negative association between Cpn presence in non-atherosclerotic segments and fibrinogen and ESR. This is an unusual finding, since Cpn infection is recognized as having a pro-inflammatory effect in atherosclerosis, alongside the rise in CRP, fibrinogen and other markers of inflammation. Filardo et al. have performed a meta-analysis of studies on Cpn-mediated inflammation in atherosclerosis, and the results showed that seropositive subjects had had higher levels of fibrinogen, hsCRP and IL-6 compared to seronegative patients [27,48].

Our study revealed an unusual but significant negative correlation between HP presence and expected glomerular filtration rate, and a positive correlation with urea and creatinine levels, which persisted after controlling for age, sex and BMI. It could be that HP infection is associated with renal dysfunction. Studies have reported a link between cytotoxin-associated gene A-positive HP and diabetic nephropathy, IgA nephropathy, membranous nephropathy and other renal disorders [51]. On the other hand, systematic reviews and meta-analyses in recent years did not confirm the effect of HP infection on chronic renal disorders, where some studies showed an inverse relationship, with renal patients having lower HP rates (probably due to HP eradication treatment effects) [52,53].

One of the possible explanations could be the fact that 19% of our subjects had a history of chronic renal disease stage 2 or 3, but this association was not seen specifically in this subpopulation. Previous studies focused on general population found no significant association of HP infection with urea and creatinine levels, eGFR [54,55]. Recently, Wang et al. [56] published their findings on the prevalence of HP infection and its association with eGFR in a Chinese population, where, just like in our study, participants with lower eGFR had a higher prevalence of HP infection, but the strength of this correlation was weakened after taking into consideration age and sex as confounders.

### Limitations

The main limitation of this study is the sample size and the lack of follow-up data on long-term clinical outcomes, but taking into consideration the specific nature of the sampling location, it was very difficult to collect even this amount of samples.

There are a few significant limitations to our study apart from the sample size. Although the presence of HP, CMV and Cpn in atherosclerotic lesions is confirmed in many studies, it would be helpful if we had the opportunity to take parallel samples from affected and non-affected arteries, as well as samples from non-CAD subjects, to support the quality of our results and maybe help us clarify some of the results.

Also, the initial CRP values in our sample were higher than expected in patients with no significant acute process. At the time of the initial consultation, none of the patients had shown or reported signs of acute bacterial/viral infection or had been using antibiotics. The higher than normal CRP values recorded in most of the samples could be influenced by several features of our sample, such as the average age (mean age around 65 years), presence of obesity, having multiple chronic disorders, use of medications, earlier COPD exacerbation, venous thrombosis or hematoma. Of course, the inflammatory component of ASCVD could be one of the culprits, as well as infections caused by HP, CMV or Cpn, as indicated in our manuscript. All of those factors have been recognized in the literature as potential non-infectious causes of elevated CRP [57].

## 5. Conclusions

It has been a long road from the first assumptions of William Osler [58] to the latest studies on the effects of HP, Cpn, CMV and other microorganisms on the initiation, progression and complications of atherosclerosis, especially CAD [59].

The preliminary results of this pilot study indicated the presence of HP, CMV and Cpn in apparently healthy arterial segments in patients with CAD, not only in coronary arteries, but in internal mammary arteries, otherwise considered resistant to the atherosclerosis. There was a significant correlation between microorganism DNA presence and inflammation markers such as fibrinogen and ESR, pointing to possible early inflammatory effects on the progression of atherosclerosis to the previously unaffected arterial segments. We also found an unexpected association between HP presence and eGFR that needs further analysis. Hopefully, all these findings will be good building blocks for further studies on the complex interaction between infectious agents and atherosclerosis.

## Figures and Tables

**Table 1:** Sequences of the primers used in PCR.

Microorganism/Gene	Primers (5′-3′)/Probe
*Chlamydia pneumoniae*	Forward—GTTGTTCATGAAGGCCTACT Reverse—TGCATAACCTACGGTGTGTT
*Helicobacter pylori*	Forward—CGCTGAAATCTCTCTTTATG) Reverse—CATGCTTTGATTGCCGATAGC
Cytomegalovirus	Forward—GAAGGTGCAGGTGCCCTG Reverse—GTCTCGACGAACGACGTACG
Glyceraldehyde 3-phosphate dehydrogenase	Forward—GGGCTCTCCAGAACATCATCC Reverse—GTCCACCACTGACACGTTGG Probe—FAM-CCTCTACTGGCGCTGCCAAGGCT-TAMRA

**Table 2:** Basic demographics of the study sample.

Variables	*n* = 56 (%)
Sex, *n* (%) male female	48 (85.7%) 8 (14.3%)
Age, years, mean ± SD	64.7 ± 8.8
BMI, kg/m^2^, mean ± SD	26.7 ± 3.6
BMI category, *n* (%) normal overweight obesity	19 (33.9%) 25 (44.6%) 12 (21.4%)
Diagnosis on admission, *n* (%) Stabile AP NSAP IM	14 (25.0%) 25 (44.6%) 17 (30.4%)
Smoking, *n* (%) smoker non-smoker	22 (39.3%) 34 (60.7%)
Stenosis, *n* (%) LM LAD CX RCA	27 (48.2%) 53 (94.6%) 46 (82.1%) 44 (78.6%)
Rhythm disorder, *n* (%) Atrial fibrillation Ventricular tachycardia/V. Fibrillation Ventricular extrasystole	11 (19.6%) 2 (3.6%) 3 (5.4%)

BMI: Body mass index; AP: Angina pectoris; NSAP: Non-stable angina pectoris; IM: Myocardial infarction; LM: Left main artery; LAD: Left anterior descending artery; CX: Circumflex artery; RCA: Right coronary artery.

**Table 3:** Laboratory parameters with used reference values.

Laboratory Parameters	Value	Reference Values and Units
CRP, median (range)	40.80 (0.50–179.40)	0–5 mg/dL
Fibrinogen, mean ± SD	4.29 ± 1.06	2–4 g/L
ESR, median (range)	23.00 (2.00–95.00)	2–12 mm/h
Total cholesterol, mean ± SD	4.53 ± 1.34	<5.2 mmol/L
HDL-C, mean ± SD	1.10 ± 0.34	>1.3 mmol/L
LDL-C, median (range)	2.31 (0.86–8.50)	<3.3 mmol/L
Triglycerides, median (range)	1.62 (0.42–4.90)	<1.7 mmol/L
Urea, mean ± SD	7.51 ± 2.48	2.8–8.3 mmol/L
Creatinine, mean ± SD	101.12 ± 32.97	62–103 umol/L
eGFR, mean ± SD	55.61 ± 9.29	>60 mL/min
Uric acid, mean ± SD	385.61 ± 130.45	142–416 umol/L
Leukocytes, mean ± SD	9.50 ± 2.67	3.9–10 × 10^9^/L
Erythrocytes, mean ± SD	4.18 ± 0.80	3.86 × 10^12^/L–5.08 × 10^12^/L (female) 4.34 × 10^12^/L–5.72 × 10^12^/L (male)
Hemoglobin, mean ± SD	126.41 ± 22.71	110–150 g/L (female) 120–160 g/L (male)
Hematocrit, mean ± SD	0.37 ± 0.07	0.36–0.47 l/L (female) 0.41–0.53 l/L (male)
Platelets, mean ± SD	202.77 ± 54.47	140–450 × 10^9^/L

CRP: C-reactive protein; ESR: Erythrocyte sedimentation rate; HDL-C: High-density lipoprotein cholesterol; LDL-C: Low-density lipoprotein cholesterol; eGFR: expected glomerular filtration rate.

**Table 4:** Linear regression analysis using presence of microorganisms as an independent variable.

Dependent Variable	HP Presence	Cpn Presence	CMV Presence
CRP	B = 24.344 *p* = 0.159	B = 24.584 *p* = 0.349	B = 7.656 *p* = 0.761
Fibrinogen	B = 0.989 *p* = 0.016 *	B = −1.509 *p* = 0.016 *	B = 1.136 *p* = 0.057
ESR	B = 7.734 *p* = 0.352	B = −37.796 *p* = 0.004 *	B = 42.571 *p* = 0.001 *
Total cholesterol	B = −0.020 *p* = 0.972	B = 0.314 *p* = 0.708	B = −0.691 *p* = 0.394
HDL-C	B = 0.252 *p* = 0.066	B = −0.252 *p* = 0.225	B = 0.070 *p* = 0.726
LDL-C	B = −0.217 *p* = 0.712	B = 0.364 *p* = 0.685	B = −0.815 *p* = 0.347
Triglycerides	B = −0.380 *p* = 0.346	B = 0.374 *p* = 0.543	B = −0.071 *p* = 0.904
Urea	B = 1.693 *p* = 0.064	B = 3.037 *p* = 0.031 *	B = −3.816 *p* = 0.005 *
Creatinine	B = 25.492 *p* = 0.055	B = −5.641 *p* = 0.777	B = −13.395 *p* = 0.485
eGFR	B = −8.887 *p* = 0.016 *	B = 2.031 *p* = 0.710	B = 5.088 *p* = 0.335

CRP: C-reactive protein; ESR: Erythrocyte sedimentation rate; HDL-C: High-density lipoprotein cholesterol; LDL-C: Low-density lipoprotein cholesterol; eGFR: Expected glomerular filtration rate; B: β (the regression coefficient); * significance *p* < 0.05.

**Table 5:** Linear regression analysis using presence of microorganisms as an independent variable, controlled for age, sex and BMI.

Dependent Variable	HP Presence	Cpn Presence	CMV Presence
CRP	B = 23.173 *p* = 0.189	B = 26.667 *p* = 0.321	B = 12.980 *p* = 0.610
Fibrinogen	B = 1.070 *p* = 0.010 *	B = −1.288 *p* = 0.039 *	B = 1.180 *p* = 0.046 *
ESR	B = 7.272 *p* = 0.396	B = −37.045 *p* = 0.006 *	B = 44.636 *p* = 0.001 *
Total cholesterol	B = −0.226 *p* = 0.681	B = 0.099 *p* = 0.906	B = −0.456 *p* = 0.568
HDL-C	B = 0.238 *p* = 0.075	B = −0.227 *p* = 0.261	B = 0.119 *p* = 0.533
LDL-C	B = −0.362 *p* = 0.542	B = 0.270 *p* = 0.766	B = −0.580 *p* = 0.502
Triglycerides	B = −0.476 *p* = 0.242	B = 0.296 *p* = 0.632	B = 0.087 *p* = 0.882
Urea	B = 1.796 *p* = 0.048 *	B = 2.914 *p* = 0.037 *	B = −4.223 *p* = 0.002 *
Creatinine	B = 26.484 *p* = 0.050 *	B = −7.377 *p* = 0.716	B = −17.143 *p* = 0.375
eGFR	B = −9.447 *p* = 0.014 *	B = 1.122 *p* = 0.844	B = 5.438 *p* = 0.318

CRP: C-reactive protein; ESR: Erythrocyte sedimentation rate; HDL-C: High-density lipoprotein cholesterol; LDL-C: Low-density lipoprotein cholesterol; eGFR: Expected glomerular filtration rate; B: β (the regression coefficient); * significance *p* < 0.05.

## Data Availability

The data presented in this study are available on request from the corresponding authors, as the data are a part of an ongoing doctoral thesis research.
